# Clustering by Errors: A Self-Organized Multitask Learning Method for Acoustic Scene Classification

**DOI:** 10.3390/s22010036

**Published:** 2021-12-22

**Authors:** Weiping Zheng, Zhenyao Mo, Gansen Zhao

**Affiliations:** 1 School of Computer Science, South China Normal University, Guangzhou 510631, China; gzhao@m.scnu.edu.cn; 2 School of Computer Science and Engineering, South China University of Technology, Guangzhou 510006, China; csmozhenyaomyz@mail.scut.edu.cn

**Keywords:** acoustic scene classification, convolutional neural network, acoustic scene clustering, multitask learning, late fusion

## Abstract

Acoustic scene classification (ASC) tries to inference information about the environment using audio segments. The inter-class similarity is a significant issue in ASC as acoustic scenes with different labels may sound quite similar. In this paper, the similarity relations amongst scenes are correlated with the classification error. A class hierarchy construction method by using classification error is then proposed and integrated into a multitask learning framework. The experiments have shown that the proposed multitask learning method improves the performance of ASC. On the TUT Acoustic Scene 2017 dataset, we obtain the ensemble fine-grained accuracy of 81.4%, which is better than the state-of-the-art. By using multitask learning, the basic Convolutional Neural Network (CNN) model can be improved by about 2.0 to 3.5 percent according to different spectrograms. The coarse category accuracies (for two to six super-classes) range from 77.0% to 96.2% by single models. On the revised version of the LITIS Rouen dataset, we achieve the ensemble fine-grained accuracy of 83.9%. The multitask learning models obtain an improvement of 1.6% to 1.8% compared to their basic models. The coarse category accuracies range from 94.9% to 97.9% for two to six super-classes with single models.

## 1. Introduction

Acoustic scene classification (ASC) refers to the task of associating a semantic label to an audio stream that identifies the environment in which it has been produced [1]. This task takes as input a relatively long sound clip and outputs predicted acoustic scene class, e.g., home, park, and bus. Classifying scenes by audio data has its unique advantages. The recording of audio data is not restricted by the camera angle and illumination condition, etc. As a result, the equipment for sound collection can be installed in a wider range where object occlusion is no more a problem. The collection can run indiscriminately in a dark environment. Moreover, the storage cost of audio data is relatively low compared to image or video data. Recently, ASC has shown huge potentials in many industrial and business applications [2,3], such as surveillance, life-logging, and advanced multimedia retrieval [4].

The inter-class similarity is a common challenge in machine learning research [5]. However, it is getting more prominent in ASC, as the labels of the scenes are commonly annotated according to the spatial functions related to the place where the audio segments are recorded. Consequently, there are audio segments that are quite similar in terms of acoustical characteristics while assigned with different labels, e.g., the segments of a library and those of an office. It is therefore a challenging task to distinguish these similar scenes even for humans and they are often misclassified by the machine learning algorithms.

Furthermore, in most cases, misclassification occurs among similar scenes. For example, in the TUT Acoustic Scenes 2017 dataset [6], the scene of the beach is misclassified in most cases as a residential area. Additionally, home is frequently misclassified as a library, and so on. These errors seem forgivable considering the similarities existing among the scenes. For example, pedestrians, laughter, blowing wind, and other sounds exist in both beach and residential areas. The scenes of home and library may have common aspects, for instance, the quietness, low-voice speaker, and phone ring. Hence, acoustic scenes tend to be misclassified as those having similar characteristics.

Based on the above, here we propose learning the similarity relations of acoustic scenes by taking advantage of the classification errors. In our method, we use the spectral clustering algorithm on the confusion matrix. The scene (class) set of a certain acoustic scene dataset is then divided into several subsets according to the similarity relation of the corresponding acoustic scenes. Each subset is assigned a super-class label. Using this approach, a two-level class hierarchy can be easily built in the label space of the acoustic scene dataset.

Ye et al. [7] proposed an acoustic event taxonomy construction approach based on between-dictionary distances. Li et al. [8] also proposed an acoustic scene clustering method using agglomerative hierarchical clustering on deep embedding extracted by Convolutional Neural Network (CNN). Their taxonomy heavily depends on the quality of acoustic feature learnt and the distance metric selected. Conversely, our construction approach is a simple solution that does not need any feature embedding.

In this paper, the two-level class hierarchy is further integrated into a multitask learning framework for ASC. To take advantage of the relevance between the super-class (coarse category) and original class (fine-grained category), a regularization method is adopted to optimize the training. Note that multitask learning is not a new idea for ASC. Tonami et al. [9] proposed a multitask learning-based solution for joint analysis of acoustic events and scenes where each sample was given both scene and event labels through manual annotation. Abrol et al. [10] also proposed a multitask model which was trained with hierarchical coarse and fine labels for ASC. They manually created a two-level class hierarchy by arranging the fine scene classes into coarse classes. In our proposed self-organized multitask learning method, the original label space is organized into a hierarchical structure by learning the similarity relationship from the confusion matrix. In our method, manual annotation is not required, and the class hierarchy is constructed automatically solely based on the original dataset. This is the reason that the proposed method is called “self-organized” multitask learning.

**Figure 1:**
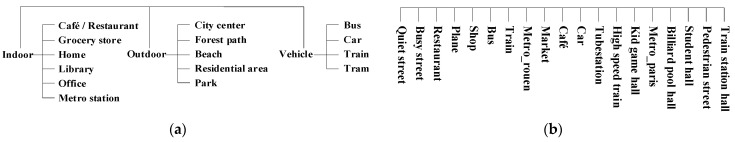
Different organizations of the label spaces. The label space of TUT Acoustic Scenes 2017 dataset is originally organized as a two-level hierarchy, however, merely single-level labels are provided in the LITIS Rouen dataset. (**a**) Labels of the TUT Acoustic Scenes 2017 dataset. (**b**) Labels of the LITIS Rouen dataset.

The proposed method is evaluated comprehensively on two publicly available datasets including the TUT Acoustic Scenes 2017, and the LITIS Rouen [11] datasets. As shown in Figure 1, there are 15 acoustic scenes and 3 super-classes in the TUT Acoustic Scenes 2017 dataset. It is arranged as a two-level class hierarchy by the original dataset publishers. We compare the constructed class hierarchy with the original one in the experiment. The LITIS Rouen dataset provides single-level classes. The experiment demonstrates that a single-level class dataset can also benefit from the proposed method.

The contributions of this paper are as the following:

(1) To the best of our knowledge, we are the first to automatically construct a taxonomy for acoustic scenes by learning similarity relationships from classification errors.
(2) By incorporating the constructed two-level class hierarchy, the proposed self-organized multitask learning method improves the performance of ASC.

The rest of the paper is organized as follows: Section 2 introduces the related works. Section 3 describes the proposed method. Experimental results and analyses are presented in Section 4. Discussion is provided in Section 5. Finally, we conclude in Section 6.

## 2. Related Works

Audio classification has become a hot topic in the field of signal processing. As an essential part of the audio classification, ASC has been one of the main tasks in the IEEE DCASE Challenges (2013, 2016–2021). In the conventional ASC techniques, cepstrum coefficients, as well as other handcrafted audio features, are classified by the Gaussian mixture models (GMM), hidden Markov models (HMM) and support vector machine (SVM) methods [12]. For example, Ma et al. [13] used a hierarchical HMM-based model fed by MFCC features to classify the environmental sounds. Chakrabarty et al. [14] further proposed a hybrid GMM–HMM system, where average modulation statistics of the scene provided by the GMM and temporal trajectories of the modulations obtained by the HMM are fused to achieve better classification performance. Recently, deep learning techniques have also achieved impressive results on acoustic scene classification [15], e.g., CNN [16,17,18,19], RNN [20], LSTM [21], DNN [22,23], and their combinations [21]. Among these models, CNN is the most popular architecture which has shown promising results in most recent works. For instance, Eghbal-Zadeh et al. [16] proposed an approach using deep CNN and binaural i-vectors [24] for ASC. Bae et al. [21] also presented a structure composed of parallel CNN and LSTM [25] networks, which aimed to extract both spectro-temporal locality and sequential information.

As in the ASC, inter-class similarity has been a serious problem for fine-grained visual recognition. To distinguish the degree of similarity among classes, the common solution is to use triplet loss [26], quadruplet loss, or N-pair-mc loss [27]. In these methods, the hierarchical relation of classes should be carefully arranged. The selections of anchor points in these methods are challenging. Nevertheless, these approaches often require a larger number of training samples and result in more complex optimizations. Multitask learning is another solution that needs hierarchical labels. Xie et al. [28] studied the large intra-class and small inter-class variance in fine-grained image classification and proposed a multitask learning framework. Zhang et al. [26] also designed a multitask framework to learn fine-grained feature representations. In their framework, hierarchy and share attributes are embedded by optimizing both classification and similarity constraints. Wu et al. [29] further formulated a multitask loss on CNN architecture to utilize the semantic relationships among food categories.

In this paper, we focus on the inter-class similarities problem in ASC and use a similar multitask learning solution as in fine-grained visual recognition. Hierarchical labels were also utilized in [10,22] for multitask learning of ASC. Nevertheless, in those works, the problem of automatic construction of class hierarchy was not considered. Recently, multitask learning approaches have been proposed to perform a joint analysis of acoustic scenes and events [9,30]. However, in these methods, the datasets should be intentionally prepared using audio synthesis or additional manual annotation.

The size of datasets in ASC is relatively small thus it may lead to over-fitting. High-quality labeled data are expensive to acquire, especially for audio data. To increase the size of the datasets, data augmentation is widely used and proven as effective practice [31,32]. Salamon et al. [32] augmented the data by deforming the audio signal directly before converting it into log-Mel spectrograms. The applied deformation included time stretching, pitch shifting, dynamic range compression, and background noise.

Nevertheless, not all augmentation techniques are helpful. Those samples augmented far from their original ones are harmful to the classification performance. To solve this problem, Lu et al. [33] proposed a metric learning-based framework to ensure appropriate augmentation for the appropriate training data. In [31], a GAN-based [34] method was used to generate additional samples for ASC. These samples were selected by an SVM hyperplane to ensure augmentation quality.

Zhong et al. [35] proposed a random erasing method for CNN data augmentation. In the method, a rectangle region is randomly selected within an image. The pixels in the region are erased with random values. This method is easy to implement, and random erasing keeps most of the information in the original image. As a result, the filtering operation to remove the harmful augmented samples performed in [31,33] is not necessary here. Gharib et al. [36] applied a similar random erasing method for ASC and achieved an improvement of 0.13 percent compared with their baseline system. 

Mixup [37] is another interesting data augmentation method. This method constructs a new example using a linear interpolation of two random examples from the training set and their labels. Xu et al. [18] used a multi-channel CNN in ASC and applied mixup to improve prediction accuracy. In this paper, a class hierarchy construction method is proposed which appends super-class labels for the training examples. The method does not increase the size of the dataset. However, it extends the label space of the samples, hence providing more information. It is demonstrated in our experiment that using multitask learning with a two-level class hierarchy can effectively enhance the generalization of the CNN model. Although both mixup and class hierarchy construction bring changes into the label space, mixup modifies the labels from the one-hot into the ratio type, whereas the class hierarchy construction provides more one-hot labels by constructing super-class labels.

## 3. Proposed Method

### 3.1. Overview

**Figure 2:**
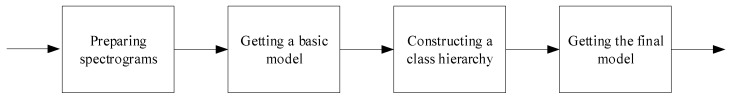
Flowchart of the proposed method.

In this paper, we propose a self-organized multitask learning method. The proposed solution includes two stages: a two-level class hierarchy is automatically constructed in the first stage using a basic model. The final classifier is then obtained by training a multitask learning model using the constructed super-class labels and the original fine-grained labels in the second stage. As shown in Figure 2, the proposed method for ASC includes the following four steps:

(1) Preparing spectrograms: transforming the raw audio segments into spectrograms that are suitable for CNN models.
(2) Getting a basic model: training a single-task CNN model as a basic model using the spectrograms and original fine-grained scene labels.
(3) Constructing a class hierarchy: testing the validation set on the basic model to obtain a confusion matrix. The spectral clustering is performed on the confusion matrix to generate super-classes.
(4) Getting the final model: training a multitask CNN model as the final classifier to predict both the original scene class and the constructed super-class using hierarchical labels.

### 3.2. Spectrograms Generation

To apply CNN models, spectrograms are generated from the audio segments using certain signal processing methods, e.g., the Short-Time Fourier Transform (STFT) [38], Constant-Q-Transform (CQT) [39], and Mel Frequency Cepstral Coefficients (MFCC) [40]. They are split into multiple samples and fed into the CNN model. The spectrogram is considered as a time-frequency representation of the acoustic scene [41]. As CNN is effective in learning spatially local correlations from images, it can use the spatial and temporal information in the spectrograms. However, different spectrograms have different processing abilities for the corresponding frequency range, which can be used for characterizing different acoustic scenes. Therefore, CNN models are widely used as the deep feature extractor, and multiple spectrogram fusions are usually applied in ASC for performance enhancement [17,42,43].

In this paper, we generate three kinds of spectrograms, STFT, CQT, and log-Mel spectrograms, and then evaluate the proposed method on these different presentations, respectively. Details about the spectrogram generation are described in Section 4.1.

### 3.3. Basic Model

Using these spectrograms with fine-grained labels, a CNN model is trained to classify the acoustic scenes. The trained CNN model is referred to as the basic model. A VGG-like network [17] is considered here as the basic model and its structure is illustrated in Table 1. After training on different spectrograms, various basic models become available. Specifically, three basic CNN models are evaluated in the paper, namely the VGG-STFT, VGG-CQT, and VGG-Log-Mel models.

Without loss of generality, the architecture of CNN and the spectrogram are not specified below. Suppose we are given a set of n training samples $TS=\left\{\left(x_{1},\;y_{1}^{o}\right),\;\ldots ,\;\left(x_{n},\;y_{n}^{o}\right)\right\}$ with $y_{i}^{o}\in \left\{1,\ldots ,C\right\}$ indicating the fine-grained acoustic scene class label of image, $x_{i}$, i∈[1,n] (namely a spectrogram patch); superscript o denotes original labels of the dataset. 

The CNN network consists of multiple convolutional and pooling layers. At the end of the network, the output layer uses a softmax activation function to assign probabilities to each possible class, where there are *C* nodes in the output layer. Let $P(y_{i}^{o}|x_{i})$ be the probability corresponding to the true-ground class of $x_{i}$. There are also L nodes in the next-to-last layer, which are mapped to *C* nodes using the fully connected layer. Let $W_{v,u}\left(v\in \left[1,C\right];u\in \left[1,L\right]\right)$ denote the weights of connections between these two layers. A negative log-likelihood loss is adopted in the basic model, i.e.,

(1) $$Loss\left(W\right)=\frac{1}{n}\sum_{\left(x_{i},y_{i}^{o}\right)\in TS}(-\log P(y_{i}^{o}|x_{i},W))+\alpha {\left|\left|W\right|\right|}_{2}^{2}$$

### 3.4. Super-Class Labels Construction

Constructing the super-classes by merging similar acoustic scenes is a natural and straightforward method. However, it is difficult to find out scenes with similar acoustical properties. Generally, the audio segments are transformed into a certain kind of embedding, and distances defined on the embedding spaces are used to group the scenes into coarse categories. However, learning of embedding and distance definition is not easy, and the clustering results are hard to explain.

**Figure 3:**
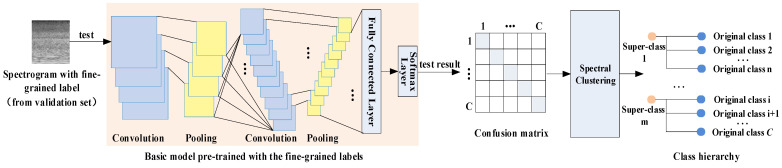
The pipeline of the construction of super-class labels. Each sample in the validation set is evaluated by the trained basic CNN model. The prediction results are collected to compute a confusion matrix. Then, a spectral clustering algorithm is performed upon this matrix. As a result, original acoustic scene classes are clustered into several subsets. The classes in the same subset are considered as similar scenes and the same super-class label is assigned to their corresponding samples.

In our research, we use misclassification information to approximate the similarities among classes. Specifically, a certain set (e.g., the validation set) of samples is evaluated on a basic model. These predicted results are counted into a confusion matrix. Finally, a spectral clustering algorithm is applied to construct super-class labels and thus expand the label space. The pipeline is illustrated in Figure 3.

For a certain basic CNN model, such as the VGG-STFT, a confusion matrix F can be calculated, with $F_{ci,\;cj}$ denoting the number of the samples of class ci that are classified as class cj by that model. After pre-processing, F is transformed to a matrix D to ensure symmetry:

(2) $$D=\left(F+F^{T}\right)/2$$

Using this matrix *D*, we apply spectral clustering [44] to divide the original C classes into N subsets $H_{1},\ldots ,H_{N}$,$\;H_{1}\cup^{\text{​}}\ldots \cup^{\text{​}}H_{N}=\left\{1,\ldots ,C\right\}$; $H_{hi}\cap^{\text{​}}H_{hj}=\emptyset$( hi≠hj;hi,hj∈[1,N]). The proposed clustering algorithm is provided in Algorithm 1. 

Each subset is assigned a super-class label. The TS can be rewritten as $TS=\left\{\left(x_{1},\;\langle y_{1}^{o},y_{1}^{e}\rangle \right),\;\ldots ,\;\left(x_{n},\;\langle y_{n}^{o},y_{n}^{e}\rangle \right)\right\}$ with $y_{i}^{e}\in \left\{1,\ldots ,N\right\}$ indicating the super-class label of $x_{i}$, i∈[1,n], where superscript e denotes the expanded label. Therefore,

(3) $$\forall i\forall j\exists m\left(\left(y_{i}^{o}\in H_{m}\wedge y_{j}^{o}\in H_{m}\right)\to y_{i}^{e}=y_{j}^{e}\right)$$

The number of super-classes in the above construction (i.e., *N*) is a hyperparameter and is selected using experiments.
**Algorithm 1.** Clustering algorithm in super-class generation**Input:** the confusion matrix F, number of clusters N. **Output:** super-class clusters $H_{1},\ldots ,H_{N}$ 1. Set the diagonal elements of F to zero: $\;F_{k,k}=0,k\in \left\{1,\ldots ,C\right\}$
2. Normalize each row of *F* by the following equations: $\;SM_{k}=\overset{C}{\underset{d=1}{\sum }}F_{k,d}$
$\;F_{k,t}=F_{k,t}/SM_{k}$, k,t∈{1,…,C} 3. Transform F into a symmetric matrix *D*: $\;D=\left(F+F^{T}\right)/2$ 4. Assume that B is a diagonal matrix whose elements are set as: $\;B_{e,e}=\overset{C}{\underset{q=1}{\sum }}D_{e,q}$, e∈{1,…,C} 5. Construct the Laplacian matrixG by:  G=B−D 6. Calculate the eigenvectors $a_{1},\;a_{2},\ldots ,a_{N}$ corresponding to the *N* smallest eigenvalues of G. 7. Let $A\in R^{CN}$ be the matrix containing $a_{1},\;a_{2},\ldots ,a_{N}$ as columns; let $r_{i}\in R^{N}$ be the i-th row of A. 8. Use K-means algorithm to cluster $\left\{r_{1},\;r_{2},\ldots ,\;r_{C}\right\}$ into *N* clusters $RC_{1},\ldots ,RC_{N}$. 9. Output clusters $H_{1},\ldots ,H_{N}$ with $H_{hi}=\{z|r_{z}\in RC_{hi}\}$, hi∈{1,…,N}.

### 3.5. Multitask Learning Model

**Figure 4:**
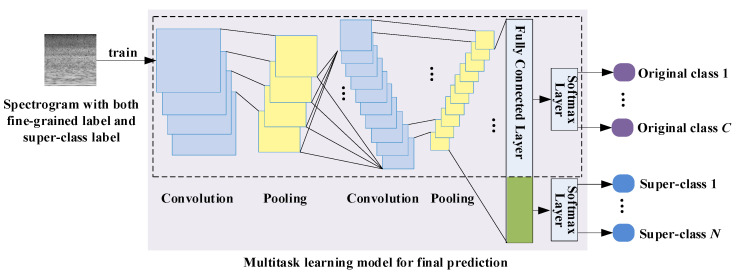
Structure of the multitask learning model. The structure within the dashed box is identical to the basic CNN model. An additional softmax layer has been added and fully connected to the next-to-last layer. The green area above represents the newly added connections in the fully connected layer. Both original acoustic scene labels and super-class labels are simultaneously predicted here.

In the second stage, the constructed two-level class hierarchy is incorporated into a multitask learning framework. The structure of the multitask learning model is illustrated in Figure 4. As it is seen the discrimination of super-class has become an additional task in the classification process. Consequently, the basic model is transformed into a multitask learning paradigm. The details are provided in the following.

To make the model aware of the inter-class similarities, we add another output layer onto the basic CNN model, leaving all other details of the model unchanged. The newly added layer has N output nodes and is fully connected onto the original next-to-last layer. The weights of the newly added connections are denoted as $U_{mj,mi}\left(mj\in \left[1,N\right];mi\in \left[1,L\right]\right)$. We then update the reconstruction error of the new model into a multitask learning form as the following:

(4) $$E=\underset{\left(x_{i},\langle y_{i}^{o},y_{i}^{e}\rangle \right)\in TS}{\sum }-(\;\gamma \log P\left(y_{i}^{o}|x_{i},W;U\right)+\left(1-\gamma \right)\log P\left(y_{i}^{e}|x_{i},W;U\right))$$

where γ∈[0,1] controls the proportion between the original task and the new task in the reconstruction error. The weight vector $W_{t}=\left(W_{t,1},\ldots ,W_{t,L}\right)$ for original class t should capture similar high-level patterns [28,45] as the weight vector for the super-class s(t) of class t, i.e., $U_{s\left(t\right)}=\left(U_{s\left(t\right),1},\;\ldots ,U_{s\left(t\right),L}\right)$. Therefore, we introduce the following regularization into the loss function:

(5) $$R=\overset{C}{\underset{t=1}{\sum }}{\left|\left|W_{t}-U_{s\left(t\right)}\right|\right|}_{2}^{2}\;\;\;\;\;\;\;\;\;\;\;\;\;\;\;\;\;\;\;\;\;$$

Finally, the loss function of the new model can be defined as:

(6) $$Loss_{ML}\left(W;U\right)=E/n+\alpha \cdot R+\beta \cdot {\left|\left|W;U\right|\right|}_{2}^{2}$$

where α and β are set to 0.0001. 

After performing the self-organized multitask learning method, respectively, we can obtain boosted models, e.g., VGG-STFT-ML (from VGG-STFT), VGG-CQT-ML (from VGG-CQT), and VGG-Log-Mel-ML (from VGG-Log-Mel). As expected, our experiments confirm that the updated model outperforms the basic models. 

Note that the CNN model is a building block in the proposed framework. It can be replaced by any other popular CNN architecture, such as ResNet [46] and GoogleNet [47]. The backbone of the multitask learning network is not necessary to be restricted by the basic model. We keep most of the layers unchanged in multitask learning model to facilitate performance comparison. 

Furthermore, the class hierarchy construction and self-organized multitask learning approaches are not limited to CNN. Hence, similar ideas apply to other models such as RNN/LSTM, DNN, and DBN, and might be suitable for other applications. 

## 4. Experiments and Results

### 4.1. Experiment Setup

The TUT Acoustic Scenes 2017 dataset [6] and LITIS Rouen dataset [11] (a revised version) are selected to evaluate the performance of our method. The TUT Acoustic Scenes 2017 dataset includes Development and Evaluation sets. We have trained the model on the Development set and evaluated it on the Evaluation set. We also follow the four-fold split provided by the dataset publishers. 

The LITIS Rouen dataset is one of the commonly used publicly available datasets for ASC. However, it tends to provide over-optimistic results as some examples cut from the same long recordings are distributed into the training set and test set, respectively. To avoid the “album effect”, Rakotomamonjy [48] had created a corrected version of the LITIS Rouen dataset, namely Rouen-15. However, it was not made public. To this end, we create a revised version by ourselves in this paper. We first merge the 3026 examples into 487 recording files according to the mapping relation presented by the dataset provider on their website. The dataset is then divided into the training set and test set by file. Similarly, four-fold cross-validation is performed. The training set is further split into four folds by file as well. At last, these files are restored into examples. Specifically, 684 examples are selected as a test set, while 2342 examples are used for training, which are split into 641, 617, 545, and 539 examples, respectively. 

Three kinds of spectrograms are generated for the evaluation experiments including STFT, CQT, and log-Mel spectrograms. Spectrograms are generated for each channel (left and right) from the audio clips. To generate STFT spectrograms, the window size is set to 16 ms (706 points) and the hop length is 9.75 ms (430 points) at 44.1 KHz for the TUT Acoustic Scenes 2017 dataset. For the LITIS Rouen dataset, the window size is 32 ms (706 points) and the hop length is 19.5 ms (430 points) at 22.05 KHz. 

Logarithmic power spectral densities ($10\log_{10}PSD$) are utilized to plot the spectrograms, which are generated in a one-sided fashion. The sizes of the spectrograms are 1024 × 354 pixels and 1537 × 354 pixels for the two datasets. The spectrograms are divided into patches with a width of 143 pixels and a shift step of 126 pixels. The size of each patch is 143 × 354 pixels. Therefore, we obtain 8 and 12 patches for each spectrogram on the two datasets, respectively.

CQT spectrograms are generated using a python library, Librosa 0.5.0. In the generation function, the sampling rate is set as 22.05 KHz, the filter scale is set to 2, and the frequency bin is 110. Other parameters are set to their default values. The CQT spectrograms with the sizes of 862 × 110 pixels and 1292 × 110 pixels are generated for the TUT Acoustic Scenes 2017 and the LITIS Rouen, respectively. The spectrograms are split into patches with a 143-pixels width and an 80-pixels shift step. Consequently, we obtain 10 and 15 patches for each spectrogram per channel on the two datasets, respectively. 

We then extract log-scaled Mel-spectrograms with 128 Mel-bands, using a window size of 92.8 ms (2048 points at 22.05 KHz) and a hop length of 46.4 ms. The sizes of the log-Mel spectrograms for the two datasets are 430 × 128 pixels and 646 × 128 pixels, respectively. The patch width is set as 143 pixels and the shift step is 71 pixels. We can generate 5 and 8 patches from each spectrogram on the two datasets. All patches are resized into 143 × 143 pixels before they are fed into the CNN networks. In addition, patches derived from both channels are separately treated as samples. Note that the above settings of hop lengths and shift steps are decided and justified in our previous work [17] and reused here for convenience.

The experiments are implemented using the TensorFlow [49] platform. A mini-batch size of 256 is used as well as an early stopping strategy with a patience parameter of 30 and a maximum epoch of 200. We use Adam [50] optimizer with a learning rate of 0.0001. In the following experiments, γ in Equation (4) is set to 0.6 for all the multitask models. An example-level majority voting accuracy is selected as a performance metric in the following experiments.

### 4.2. Selection of Super-Class Number

**Figure 5:**
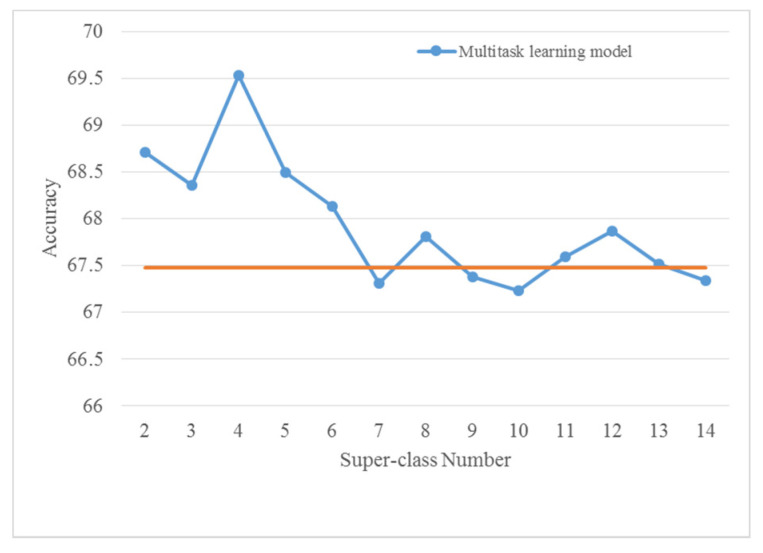
Accuracies of multitask learning models with different super-class numbers using CQT spectrograms on the TUT Acoustic Scenes 2017 dataset. The orange horizontal line means the accuracy of the basic model (VGG-CQT).

**Figure 6:**
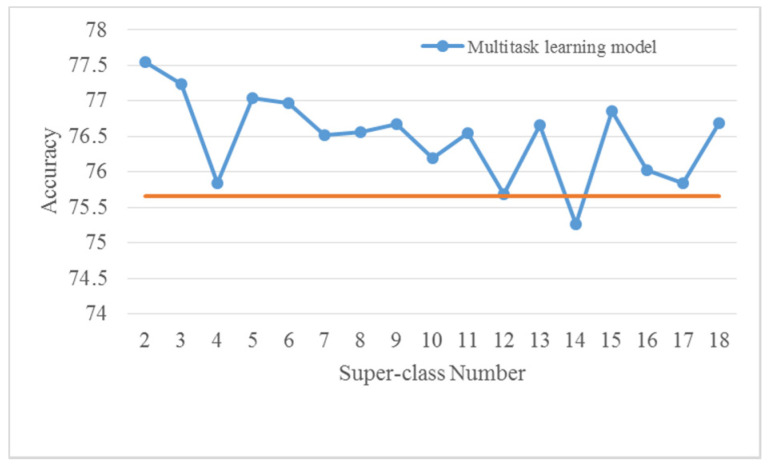
Accuracies of multitask learning models with different super-class numbers using CQT spectrograms on the LITIS Rouen dataset. The orange horizontal line means the accuracy of the basic model (VGG-CQT).

In the self-organized multitask learning models, the number of super-class is an important parameter that is closely related to the performance. For a given dataset with *C* classes, the maximum and the minimum super-class number is *C*-1 and 2, respectively. One way is to train and test the multitask learning models with all the possible super-class numbers and select the model with the highest accuracy. This, however, causes substantial waste of computing resources. To observe the differences, we have implemented the multitask learning model using CQT spectrograms on both datasets with all super-class numbers. Their corresponding accuracies are presented in Figure 5 and Figure 6.

As it is seen in Figure 5, applied on the TUT Acoustic Scenes 2017 dataset the previous five models (with super-class numbers 2, 3, 4, 5, and 6) achieve higher accuracies than that of other models. In other words, the multitask learning model with a very large super-class number is not competitive in performance. Similarly, in Figure 6, the previous five models (except for the one with 4 super-classes) provide higher accuracies on the LITIS Rouen dataset. Although the decline of performances with the increase in super-class numbers is not apparent, the best results are still achieved with relatively small super-class numbers. Consequently, only the multitask learning models with two to six super-classes are explored in the following experiments.

Note that in Figure 5 and Figure 6, it can be seen that the accuracies of the multitask learning models outperform the basic model in most cases.

### 4.3. Evaluations on the TUT Acoustic Scenes 2017 Dataset

**Figure 7:**
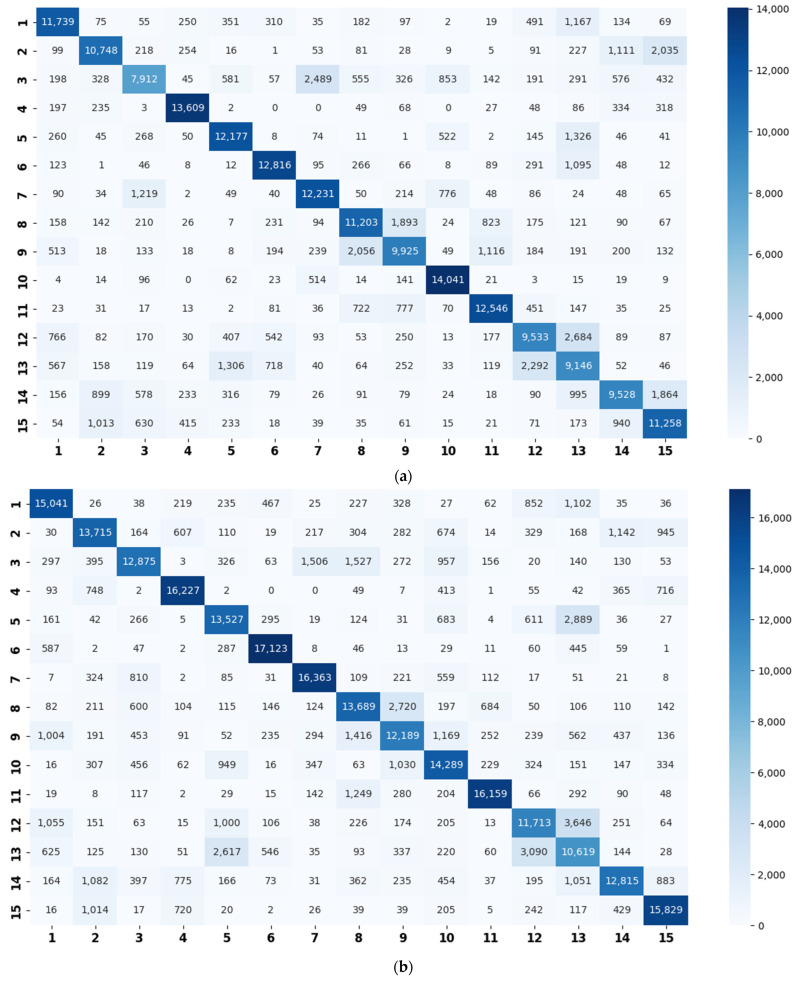

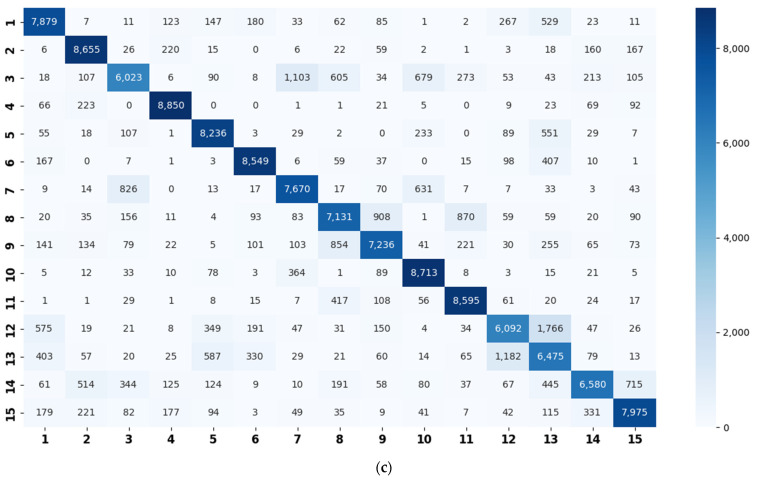
Confusion matrices on the TUT Acoustic Scenes 2017 dataset generated by the basic models (**a**) using STFT spectrograms; (**b**) using CQT spectrograms; and (**c**) using log-Mel spectrograms. The abscissa represents the predicted label, and the ordinate indicates the true label. Note: Beach (1), bus (2), café/restaurant (3), car (4), city center (5), forest path (6), grocery store (7), home (8), library (9), metro station (10), office (11), park (12), residential area (13), train (14), and tram (15).

Using the proposed method, the basic CNN models are trained on the STFT, CQT, and log-Mel spectrograms. Based on a specific basic model, a confusion matrix is generated for each validation set. To obtain more stable divisions, we repeat the process three times. The final confusion matrix used is calculated using the sum of the twelve confusion matrices (three times × four splits), as shown in Figure 7.

Based on the confusion matrices, the original 15 acoustic scenes are grouped into two to six super-classes. The division details are listed in Table 2. For example, using the confusion matrix generated by the STFT basic model (Figure 7a), the 15 acoustic scenes can be divided into two super-classes: the classes of bus, car, train, and tram are grouped as one super-class (represented as the blue squares); the other 11 classes are grouped as another super-class (represented as the red circles). Compared with the original super-classes (namely Indoor, Outdoor, and Vehicle categories, see Figure 1a), there are some interesting findings with the constructed divisions. First, for the two super-classes’ divisions, each of them keeps one original super-class and merges the other two into another super-class. For the three super-classes’ divisions, the results for CQT and log-Mel model are identical to the original Indoor, Outdoor, and Vehicle divisions. The four super-classes’ divisions for STFT, CQT, and log-Mel models are the same. In addition, the five super-classes’ divisions for CQT and log-Mel models are also identical. In fact, in most cases, the divisions are very similar to each other. The above results confirm the robustness of the class hierarchy construction method. 

In general, we rate the divisions for the log-Mel model as the best divisions. For example, its three super-classes’ division is identical to the original division, where the two super-classes’ division merges the Outdoor and Vehicle into one super-class, which seems more reasonable. It is believed that the superiority in divisions is due to the high performance of the log-Mel basic model (see Table 3). Hence, the basic classifier and the evaluated samples for confusion matrix creation should be well-chosen.

To demonstrate the effectiveness of the proposed multitask learning method, the performances of the multitask models with different super-class numbers using different spectrograms are given in Table 3. The super-class division used in the experiments here is presented in Table 2. The experiments are carried out three times and the results are then reported using the average and standard deviation in percentage. For the STFT models, the best-achieved accuracy is 62.0% obtained by the multitask model with three super-classes. This confirms an improvement of 2.0% in comparison with the basic model. Similarly, an accuracy of 69.5% is achieved by the multitask CQT model with four super-classes which is equivalent to an improvement of 2.0% over the basic model. 

The accuracy of the multitask CQT model with three super-classes is 68.4%, which is identical to the original Indoor, Outdoor, and Vehicle division. As we can see, the performance of the model using original manually grouped division is outperformed by the one using super-classes generated by the proposed method. This means that even the datasets with hierarchical labels benefit from the proposed method. 

The same situation can be observed in the log-Mel models. The accuracy of the multitask log-Mel model using three super-classes (they are the same as the original division) is 72.1%. However, the best accuracy among the multitask log-Mel models is 72.8%. This indicates an improvement of 3.5% over the basic model. All the multitask models (with two to six super-classes) have outperformed their corresponding basic models in Table 3.

A one-sided paired *t*-test was applied to obtain the statistical significant difference of the accuracy of 15-scenes between the basic model and the corresponding best-performed multitask model. The results revealed the statistical significance (significance level < 0.05) of the accuracy improvement on the STFT, CQT, and log-Mel models, respectively.

### 4.4. Evaluations on the LITIS Rouen Dataset

**Figure 8:**
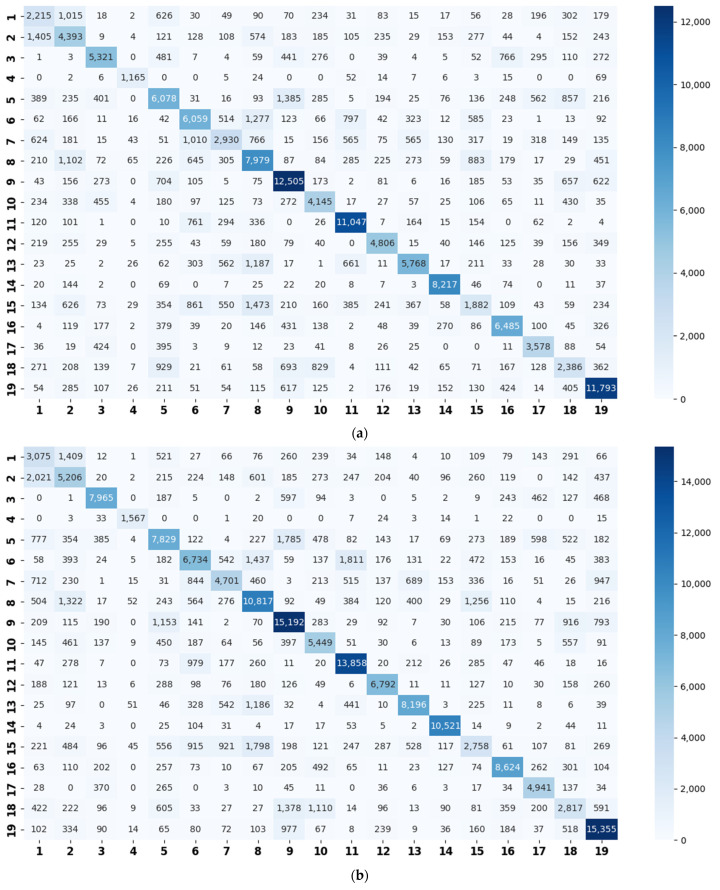

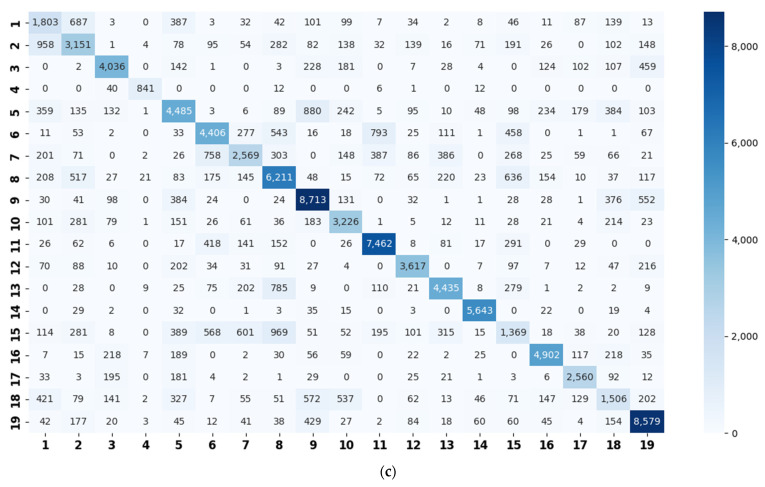
Confusion matrices on the LITIS Rouen dataset generated by the basic models (**a**) using STFT spectrograms; (**b**) using CQT spectrograms; and (**c**) using log-Mel spectrograms. The abscissa represents the predicted label, and the ordinate indicates the true label. Note: Quiet street (1), busy street (2), restaurant (3), plane (4), shop (5), bus (6), train (7), metro Rouen (8), market (9), café (10), car (11), tubestation (12), high speed train (13), kid game hall (14), metro Paris (15), billiard pool hall (16), student hall (17), pedestrian street (18), and train station hall (19).

The confusion matrix used is similarly calculated using the sum of the twelve confusion matrices generated from the validation sets (Figure 8).

According to the above confusion matrices, the 19 scene classes in the LITIS Rouen dataset are grouped into two to six super-classes, as shown in Table 4. The divisions indicate the following findings: First, it is found that the outputs of the class hierarchy construction method are stable and robust. For example, the three super-classes’ divisions for the STFT model and log-Mel model are the same. Likewise, the five super-classes’ divisions and the six super-classes’ divisions for the STFT model and CQT model, respectively, are identical. The two super-classes’ divisions and four super-classes’ devisions for the STFT model and log-Mel model are very similar as well. The difference only lies in the division of a single class. Second, the classes bus, train, metro Rouen, car, high-speed train, and metro Paris are grouped into one super-class in almost all cases. It is the equivalent of the Vehicle category in the TUT Acoustic Scenes 2017 dataset. However, the class plane is separated from the Vehicle super-class and divided as a one-element super-class, which seems more reasonable, as the plane is a kind of non-ground transportation. The classes restaurant, billiard pool hall, and student hall are also clustered as a fixed combination regularly. The features they have in common include their medium-sized indoor space and people’s close-talk. These features may produce similar acoustical characteristics. 

Table 5 compares the accuracies of basic models, as well as their corresponding multitask learning models. The experiments are repeated three times and the average and standard deviation accuracies are provided. For the STFT models, the best accuracy is 76.0% which is achieved by the multitask model with four super-classes. It is equivalent to an improvement of 1.6% over the basic model. Additionally, the five multitask models all outperform the basic one. For the CQT models, the multitask model with two super-classes achieves the best accuracy, providing an improvement of 1.9% over the basic model. Again, the five multitask CQT models are all superior to the basic model. For the log-Mel models, the best accuracy of 78.1% is achieved by the multitask model with two super-classes. This is equivalent to an improvement of 1.8% over the basic one. Similarly, the results of the *t*-test had revealed the statistical significance (significance level < 0.05) of the accuracy improvement by the corresponding best-performed multitask model (over the basic model) on the STFT, CQT, and log-Mel models, respectively. According to the results, the classification performance of LITIS Rouen dataset has been significantly improved by constructing super-classes and integrating them into the multitask learning framework. Consequently, we can see that the proposed self-organized multitask learning method is also helpful for the acoustic scene datasets with single-level labels. According to the extended super-class labels for each sample, the super-class results predicted by the multitask learning models are also evaluated and shown in Table 5. High accuracies have been achieved on the super-class classification tasks. For instance, for the log-Mel models, an accuracy of 97.9% is achieved for the two super-classes classification and it is 94.9% for the six super-classes classification.

### 4.5. Ensemble Results

Late fusion ensemble is commonly used in the domain of ASC (see, e.g., [31,51]). For instance, in [31], the linear logistic regression was performed on the classification scores of eight models to obtain fusion results by the winner of first place in the DCASE Challenge 2017. Here, we also use a late fusion ensemble. Specifically, the best multitask models are selected and combined with a simple majority voting scheme. For the TUT Acoustic Scenes 2017 dataset, these models include the multitask STFT model with three super-classes, the multitask CQT model with four super-classes, and the multitask log-Mel model with five super-classes. For the revised version of the LITIS Rouen dataset, these models include the multitask STFT model with four super-classes, the multitask CQT model with two super-classes, and the multitask log-Mel model with two super-classes. The ensemble results, as well as the state-of-the-art results for the two datasets, are displayed in Table 6.

As shown in Table 6, the ensemble result using the three best multitask models for the TUT Acoustic Scenes 2017 is 81.4%. This is higher than the accuracies of most of the state-of-the-art techniques listed in Table 6 except for the model in [31] which used GAN data augmentation. The ensemble result using the three best multitask models for our revised version of LITIS Rouen is 83.9%. For a rough comparison, the accuracy of Rouen-15 (81.8%) [48] is listed here. This is as the evaluated datasets are different. To comprehensively evaluate the multitask learning method, the three basic models are also late fused using the same ensemble method. The ensemble result on the TUT Acoustic Scenes 2017 dataset is 77.8% and the one on the LITIS Rouen dataset is 78.1%. Hence, our proposed ensemble results outperform the corresponding basic ensemble results by 3.6% and 5.8%, respectively, on the two considered datasets. 

## 5. Discussion

### 5.1. Similarity Relationship of Acoustic Scenes

The experiment results confirm our assumption that the similarity relation amongst the acoustic scenes can be reflected by the classification errors. For example, according to the confusion matrices (Figure 7), in most cases, the classes of beach, city center, and park are, respectively, misclassified as residential areas (for example, there are 1167 beach samples misclassified as residential areas in Figure 7a). Additionally, the residential areas are misclassified as parks; grocery stores are misclassified as cafés; and homes are misclassified as libraries and vice versa. It is also seen that a train is misclassified as a tram, a car is misclassified as a bus, and so on. Similarly, for the LITIS Rouen dataset (Figure 8), in most cases, the class of shop is misclassified as market; metro Rouen is misclassified as metro Paris, and the train station hall is misclassified as a market. The above scenes are similar and understandable from the human point of view, hence seem convincing to us that the similarity relationship among scenes can be learnt from the confusion matrix.

### 5.2. Advantages of the Super-Class Construction Method

Deviating from the classical acoustic scene/event taxonomy methods [7,8], the proposed super-class construction method only depends on the classification results by a basic classifier. It does not need any feature embedding. The method is simple and effective. As shown in Table 2 and Table 3, identical class hierarchies can be achieved by using classifiers on different spectrograms. Furthermore, the method does not limit the type of basic classifier. It can be SVM, random forest, and other models, although the CNN model is applied in this paper. In this sense, the proposed method has good robustness and general applicability. On the other hand, the super-class is constructed based on the similarity relations among scenes. It is more explainable compared to the embedding-based results. Finally, the proposed method is also capable to construct a multi-level class hierarchy. 

Although the construction method is proposed for ASC, it can be easily extended into acoustic event clustering and other audio taxonomy. 

### 5.3. Foundation of Self-Organized Multitask Learning

As shown in the experiments, the proposed self-organized multitask learning method can improve the ASC performance compared to the corresponding basic models. The achieved improvement comes in three ways. First, the constructed super-class labels provide more information in supervised learning. Second, teaching the model to classify the fine-grained category along with the coarse category accords with the cognitive law of “learning the easy things first” [56]. Third, according to [57], the performance of the harder task can be improved by using the information obtained from easier tasks, where predicting the super-class is an easier task. As shown in Table 5, high super-class accuracies are achieved by our models. For example, the two to six super-classes accuracies of multitask learning models using log-Mel spectrograms in the LITIS Rouen dataset are 97.9%, 97.9%, 95.6%, 95.6%, and 94.9%, respectively. Consequently, to keep the auxiliary task easy, the number of super-class should not be too large. This also justifies why competitive results are not obtained by the models with larger super-class numbers (see Figure 5 and Figure 6). 

### 5.4. Regularization by Similarity Relation

The relevance between super-class and original class is expressed as a regularization item in the multitask learning loss function (see Equation (5)). According to our experiments, this regularization slightly improves the performance. The evaluation experiments are only performed on the multitask learning models with three super-classes using STFT, CQT, and log-Mel spectrograms in both datasets. The improvements in the TUT Acoustic Scene 2017 dataset are 0.3%, 0.6%, and 0.8% and those in the LITIS Rouen dataset are 0.4%, 0.4%, and 0.6%, for STFT, CQT, and log-Mel models, respectively.

## 6. Conclusions

In this paper, the similarity relation among acoustic scenes is utilized to construct a two-level class hierarchy. The class hierarchy is further incorporated into a self-organized multitask learning framework. The experimental results show that the proposed multitask learning method can improve the classification performance effectively using different spectrograms. 

The best improvements of the STFT, CQT, and log-Mel multitask models over their corresponding basic models are 2.0%, 2.0%, and 3.5%, respectively, on the TUT Acoustic Scenes 2017 dataset. Corresponding to two to six super-classes, respectively, the coarse category classification accuracies range from 93.1 to 77.0% for STFT models, from 96.2% to 84.0% for CQT models, and from 94.9% to 85.2% for log-Mel models. By applying the late fusion strategy, a fine-grained category accuracy of 81.4% is achieved on the dataset. On the LITIS Rouen dataset, the best improvements over the corresponding basic models are 1.6%, 1.9%, and 1.8% for the STFT, CQT, and log-Mel multitask models, respectively. The super-class accuracies range from 97.1% to 95.2%, from 97.7% to 95.1%, and from 97.9% to 94.9% corresponding to two to six super-classes, for STFT, CQT, and log-Mel models, respectively. An ensemble fine-grained category of 83.9% is achieved. 

According to the experiments, the following conclusions can be drawn:

(1) The similarity relation based class hierarchy construction method is effective and reasonable.
(2) The constructed class hierarchy can be utilized to improve the ASC performance effectively in multitask learning.
(3) For a hierarchically arranged dataset, there may exist a hierarchy that is automatically constructed by our method. This may perform better than the original hierarchy in ASC.
(4) In self-organized multitask learning, the number of super-class should be chosen carefully. The multitask models with large super-class numbers would not obtain competitive results.
(5) The relevance between coarse and fine-grained classes can be utilized as regularization to improve the ASC performance.
(6) By arranging the class hierarchy, the self-organized multitask learning method provides a feasible way to promote the performance of a certain model.

In future work, we will extend this confusion matrix based super-class construction method into the domain of acoustic event taxonomy. Furthermore, to improve the performance of scene identification, the multimodal fusion method will be explored. Specifically, image and sensor data, etc., can be employed to enhance the audio data in the scene identification task.

## Figures and Tables

**Table 1:** Architecture of the proposed CNN network.

Layer	Conv1	Conv2	Pool1	Conv3	Conv4	Pool2	Conv5	Conv6	Conv7	Conv8	Pool3	Conv9	Conv10	Conv11	Full1
Kernel	5 × 5	3 × 3	Max, 2 × 2	3 × 3	3 × 3	Max, 2 × 2	3 × 3	3 × 3	3 × 3	3 × 3	Max, 2 × 2	3 × 3	1 × 1	1 × 1	−
Stride	2	1	2	1	1	2	1	1	1	1	2	1	1	1	−
Padding	2	1	0	1	1	0	1	1	1	1	0	0	0	0	−
Number of Channels	32	32	32	64	64	64	128	128	128	128	128	512	512	C	C
Dropout rate	−	−	0.3	−	−	0.3	−	−	−	−	0.3	0.5	0.5	−	−
Activation	ReLu	ReLu	−	ReLu	ReLu	−	ReLu	ReLu	ReLu	ReLu	−	ReLu	ReLu	ReLu	−
Batchnorm	Yes	Yes	−	Yes	Yes	−	Yes	Yes	Yes	Yes	−	Yes	Yes	Yes	−

Note: Symbol C in the last two columns represents the number of classes.

**Table 2:** Clustering details of the constructed super-class for the TUT Acoustic Scenes 2017 dataset. The classes marked by the same shape with the same color are grouped into the same super-class.

Clustering Scheme	Supper-Class for STFT Model	Supper-Class for CQT Model	Supper-Class for Log-Mel Model
Two Super-Classes	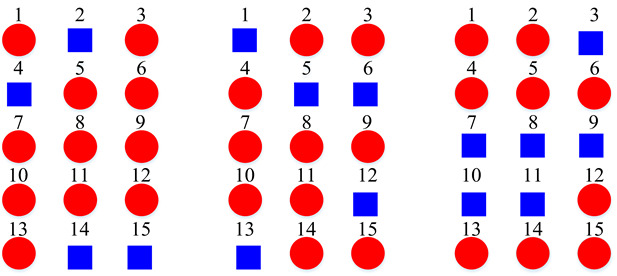		
Three Super-Classes	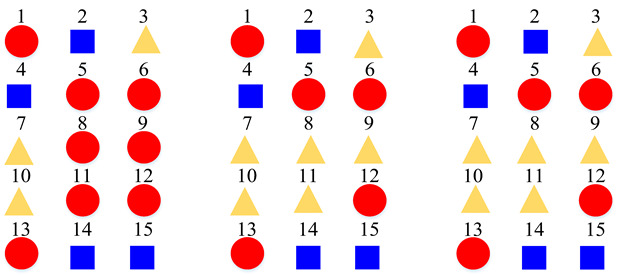		
Four Super-Classes	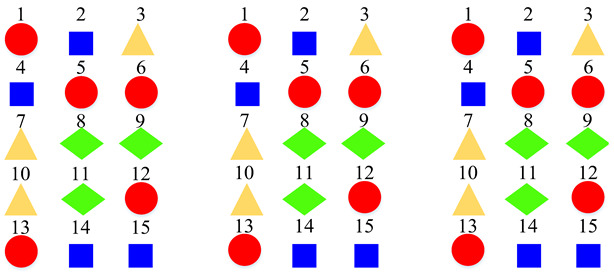		
Five Super-Classes	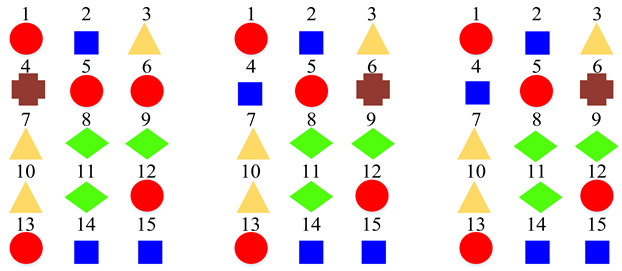		
Six Super-Classes	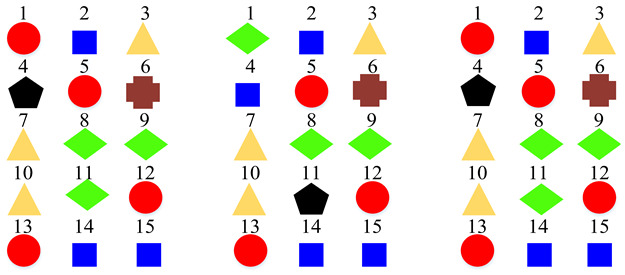		

**Table 3:** Classification performance of the TUT Acoustic Scenes 2017 dataset using different models.

Model Type	Feature Type	Super-Class Number	15 Scenes Accuracy	Super-Class Accuracy
Basic Model	STFT	/	60.0 ± 0.5	/
Multitask	STFT	2	61.1 ± 0.3	93.1 ± 0.6
Multitask	STFT	3	62.0 ± 0.1	88.8 ± 0.4
Multitask	STFT	4	61.1 ± 0.1	85.9 ± 0.8
Multitask	STFT	5	61.5 ± 0.5	82.6 ± 0.2
Multitask	STFT	6	60.7 ± 0.2	77.0 ± 0.2
Basic Model	CQT	/	67.5 ± 0.2	/
Multitask	CQT	2	68.7 ± 0.2	96.2 ± 0.1
Multitask	CQT	3	68.4 ± 0.3	92.5 ± 0.4
Multitask	CQT	4	69.5 ± 0.8	89.3 ± 0.7
Multitask	CQT	5	68.5 ± 0.4	84.8 ± 0.4
Multitask	CQT	6	68.1 ± 0.6	84.0 ± 0.6
Basic Model	log-Mel	/	69.3 ± 0.1	/
Multitask	log-Mel	2	71.5 ± 1.0	94.9 ± 0.1
Multitask	log-Mel	3	72.1 ± 0.4	93.8 ± 0.1
Multitask	log-Mel	4	71.5 ± 1.5	91.0 ± 0.4
Multitask	log-Mel	5	72.8 ± 0.7	89.5 ± 0.5
Multitask	log-Mel	6	71.9 ± 1.1	85.2 ± 0.8

**Table 4:** Clustering details of the constructed super-class for the LITIS Rouen dataset. The classes marked by the same shape with the same color are grouped into the same super-class.

Clustering Scheme	Generated by STFT Model	Generated by CQT Model	Generated by Log-Mel Model
Two Super-Classes	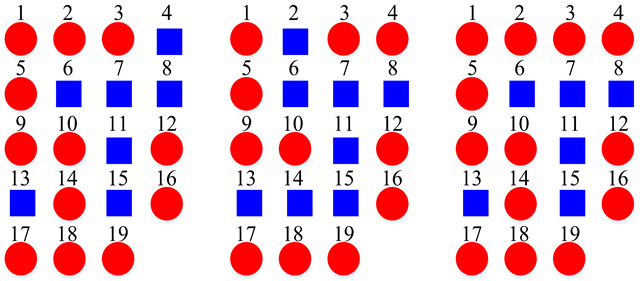		
Three Super-Classes	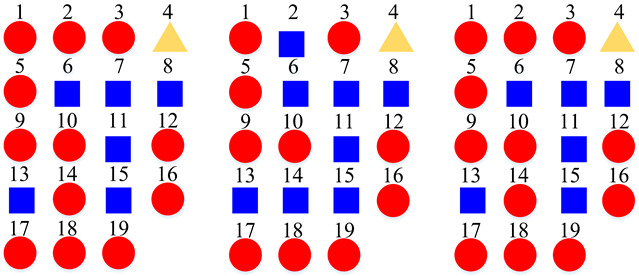		
Four Super-Classes	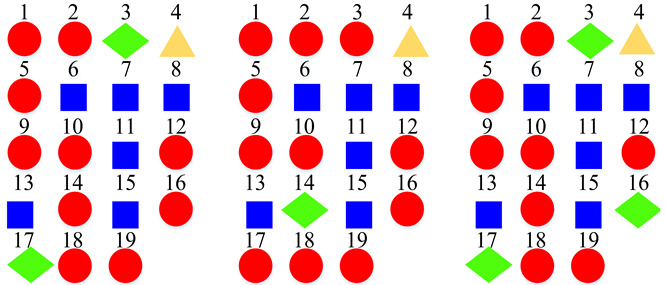		
Five Super-Classes	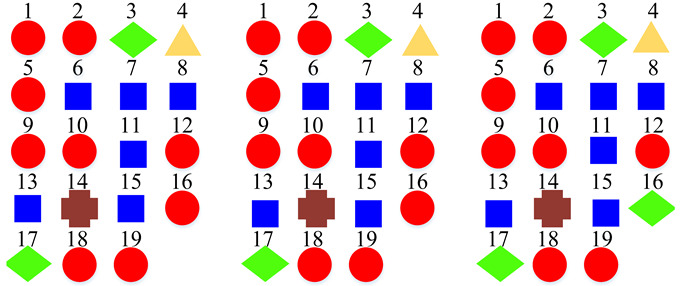		
Six Super-Classes	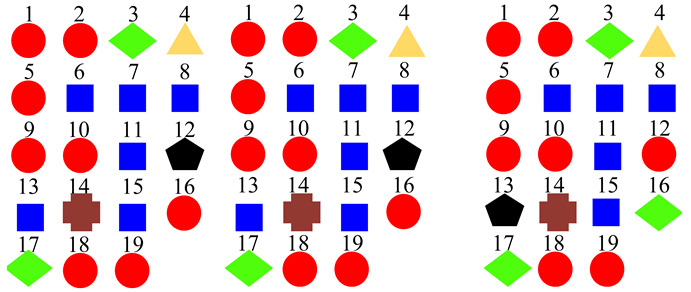		

**Table 5:** Classification performance of the revised version of the LITIS Rouen dataset using different models.

Model Type	Feature Type	Super-Class Number	19 Scenes Accuracy	Super-Class Accuracy
Basic Model	STFT	/	74.4 ± 0.7	/
Multitask	STFT	2	75.7 ± 0.6	97.1 ± 0.2
Multitask	STFT	3	75.0 ± 0.6	96.6 ± 0.2
Multitask	STFT	4	76.0 ± 0.4	95.8 ± 0.3
Multitask	STFT	5	75.5 ± 0.9	96.1 ± 0.1
Multitask	STFT	6	74.7 ± 0.5	95.2 ± 0.3
Basic Model	CQT	/	75.6 ± 0.8	/
Multitask	CQT	2	77.5 ± 0.4	97.3 ± 0.1
Multitask	CQT	3	77.2 ± 0.5	97.7 ± 0.4
Multitask	CQT	4	75.8 ± 0.7	96.6 ± 0.0
Multitask	CQT	5	77.0 ± 0.3	96.7 ± 0.2
Multitask	CQT	6	76.5 ± 0.6	95.1 ± 0.3
Basic Model	log-Mel	/	76.3 ± 0.7	/
Multitask	log-Mel	2	78.1 ± 0.3	97.9 ± 0.3
Multitask	log-Mel	3	77.7 ± 0.9	97.9 ± 0.0
Multitask	log-Mel	4	76.2 ± 0.7	95.6 ± 0.3
Multitask	log-Mel	5	76.7 ± 0.3	95.6 ± 0.2
Multitask	log-Mel	6	77.1 ± 1.0	94.9 ± 0.4

**Table 6:** Comparison of accuracies with state-of-the-art works.

Reference	Method	Accuracy	Dataset
[31]	GAN + SVM + FCNN	83.3	TUT
[51]	Background subtraction	80.4	TUT
[52]	Late fusion of CNN and ensemble classifiers	80.0	TUT
[53]	Embedded filters + DCT-based temporal module	79.2	TUT
[17]	Multi-spectrogram fusion	77.7	TUT
[18]	Mixup + multi-channel	76.7	TUT
[54]	Sound texture enhancement	75.7	TUT
[55]	Multi-spectrogram encoder-decoder	72.6	TUT
Ensemble of three basic models	Ensemble	77.8	TUT
Our method	Super-class construction + multitask learning	81.4	TUT
[48]	Supervised nonnegative matrix factorization	81.8	Rouen-15
Ensemble of three basic models	Ensemble	78.1	Rouen-revised
Our method	Super-class construction + multitask learning	83.9	Rouen-revised

Note: TUT is referred to the TUT Acoustic Scene 2017 dataset (evaluation set); Rouen-15 for the LITIS Rouen-15 dataset; and Rouen-revised for the LITIS Rouen dataset (revised version).
